# The Mediating Role of Psychological Distress in Excessive Gambling among Young People: A Four-Country Study

**DOI:** 10.3390/ijerph18136973

**Published:** 2021-06-29

**Authors:** Ilkka Vuorinen, Atte Oksanen, Iina Savolainen, Anu Sirola, Markus Kaakinen, Hye-Jin Paek, Izabela Zych

**Affiliations:** 1 Faculty of Social Sciences, Tampere University, 33014 Tampere, Finland; ilkka.vuorinen@tuni.fi (I.V.); iina.savolainen@tuni.fi (I.S.); 2 Department of Social Sciences and Philosophy, University of Jyväskylä, 40014 Jyväskylä, Finland; anu.r.s.sirola@jyu.fi; 3 Institute of Criminology and Legal Policy, University of Helsinki, 00014 Helsinki, Finland; markus.kaakinen@helsinki.fi; 4 Department of Advertising and Public Relations, Hanyang University, Ansan 15588, Korea; hjpaek@gmail.com; 5 Department of Psychology, University of Coordoba, 14004 Coordoba, Spain; izych@uco.es

**Keywords:** excessive gambling, psychological distress, loneliness, sense of mastery, adolescents, young adults

## Abstract

Background and aims: Loneliness and a low sense of mastery are associated with excessive gambling, but the underlying processes of these relationships remain unstudied. Because psychological distress can increase vulnerability to excessive gambling, we investigated its mediating role in these relationships among young people. To meet the need for cross-country research, we also observed how these relationships occur in four countries with different cultures. Design, setting, and participants: Demographically balanced cross-sectional survey data were collected from 15–25-year-olds in Finland (*n* = 1200; 50% male), the United States (*n* = 1212; 49.8% male), South Korea (*n* = 1192; 49.6% male), and Spain (*n* = 1212; 51.2% male). Measurements: Excessive gambling was measured with the South Oaks Gambling Screen, psychological distress was assessed with the 12-item General Health Questionnaire, loneliness was measured with the three-item Loneliness Scale, and low sense of mastery was assessed with the Pearlin Mastery Scale. Associations were examined first using zero-inflated negative binomial regression analyses with excessive gambling as the outcome. In addition, path analyses were performed to study how loneliness and low sense of mastery relate to excessive gambling, with psychological distress as the mediating variable. Findings: Loneliness and low sense of mastery were associated indirectly with excessive gambling via psychological distress in all country samples. Low sense of mastery was also directly associated with excessive gambling. There was a direct association between loneliness and excessive gambling only in samples from South Korea and Spain. Conclusions: Psychological distress is an important factor in understanding how loneliness and sense of mastery relate to gambling.

## 1. Introduction

There is a growing concern worldwide regarding gambling as a potential source of harm. For example, in the United States, a group of researchers recently signed a call for the gambling industry, stakeholders, and the federal government to take more responsibility so problems related to gambling can be minimised with properly scaled prevention, treatment, and recovery [1]. Global gambling expenditures have risen to hundreds of billions of euros transferred from consumers to the industry each year, whereas individuals, families, and communities tend to experience multiple financial, mental, and social problems because of excessive gambling [2]. Moreover, despite age restrictions and other limitations placed on underage gambling, positive social portrayal and technological advances have made gambling popular and even accessible among adolescents [3,4,5].

One of the most notable dangers of gambling is that it can become excessive in a way comparable to substance-related addictions [6,7,8]. Because most research concentrates on harms that are expected to be caused by excessive behaviours, situational and sociocultural risk factors have received less attention. However, some evidence suggests that people with underlying psychosocial problems related to mental health and self-regulation are more vulnerable to excessive behaviours [9,10]. In this study, we adopted the latter assumption, identifying risk factors that predict excessive gambling.

The healthy functioning of individuals depends on their psychosocial well-being and the integration of experiences through social interaction [9,11,12]. In particular, interpersonal traumas and maltreatment in childhood are linked to later distress, poor integration into society, and addictive behaviours [13,14]. Similarly, comorbidity of other excessive behaviours or psychological distress is commonly associated with excessive gambling [2,15,16,17,18,19]. For example, Ciccarelli et al. [17] found that poor decision-making and negative affective states of depression, anxiety, and stress increased the likelihood of pathological gambling. Although it is not the only pathway to excessive gambling, Blaszczynski and Nower [10] recognized that psychological distress can increase vulnerability to excessive gambling. Based on prior research findings, their model suggests that psychological distress contributes specifically to emotional vulnerability towards problem and pathological gambling. Thus, emotionally vulnerable individuals are likely to gamble to alleviate aversive affective states. However, more up-to-date research is needed to examine the role of psychological distress in diverse and non-clinical samples.

Loneliness plays an important role in excessive behaviours. For example, recreational drug use is higher among the lonely [20] and problem gamblers experience more loneliness [21,22]. In addition, a great deal of research has associated loneliness with Internet and digital addiction [21,23,24,25]. Loneliness is a subjective feeling in which one’s social relationships are qualitatively or quantitatively deficient [26]. Loneliness is an unpleasant experience and chronic loneliness due to prior experiences of isolation can be a major source of psychological distress [11,27]. Different social normative environments might increase the risk of loneliness for different reasons with a lack of satisfying relationships being more probable in stricter cultures and physical isolation being more probable in lenient cultures [28].

One possible explanation for excessive behaviours such as gambling lies in the predisposition towards being controlled by extrinsic factors. This predicts poor personal well-being, as people focus on external cues and fail to self-regulate [12]. As Orford [29] pointed out, power and powerlessness lie at the core of addictions, as industries and their stakeholders tend to profit at the expense of vulnerable populations. Sense of mastery refers to psychological resources that help individuals cope with difficult life situations [30]. High mastery means that an individual has control over their life and has the means to implement positive adaptation strategies [31]. Earlier research has related low sense of mastery to increased psychological distress [32,33,34] and excessive gambling [35]. Thus, high sense of mastery might help people cope with life stressors, whereas people with low sense of mastery might resort to excessive behaviours to cope with these kinds of stressors.

This article is focused on the mediating role of psychological distress in excessive gambling among 15–25-year-olds in Finland, the United States, South Korea, and Spain, with loneliness and sense of mastery as the predicting variables. Excessive gambling in young people is a global phenomenon, but there is a need for cross-country research investigating potentially related psychosocial factors [10,11,21]. This study’s cross-country design facilitated the comparison of the same psychological phenomenon in culturally different settings. Finland, the United States, South Korea, and Spain are also geographically distinct, as they represent Nordic and southern European nations and larger Western and Eastern cultures. These countries share similarities in their gambling prevalence rates [36], although they differ in gambling laws and regulations.

Prior literature suggests that psychological distress increases the likelihood of excessive gambling [10,17], and individuals who experience loneliness [12,13,14,15,16] or have lower sense of mastery [28] are also more vulnerable to excessive behaviour. Furthermore, loneliness and low sense of mastery are related to higher psychological distress [25,26,27]. Based on the literature reviewed, our main research hypothesis was that psychological distress mediates the relationship between loneliness, sense of mastery, and excessive gambling. We also investigated differences in the relationships between the independent variables and excessive gambling in the four countries included in this study. In accordance with previous research, we expected that gambling behaviour among young people would be similar in different countries [37].

## 2. Methods

### 2.1. Participants

The sample consisted of 4816 young people aged 15–25 years, of whom 1200 were from Finland (mean 21.29, SD 2.85; 50% male), 1212 were from the United States (mean 20.05, SD 3.19; 49.8% male), 1192 were from South Korea (mean 20.61, SD 3.24; 49.6% male) and 1212 were from Spain (mean 20.07, SD 3.16; 51.2% male). All samples were demographically balanced in terms of age, gender, and living area. The samples were based on the general populations of the four countries and were not chosen on the basis of excessive gambling engagement. This allowed us to examine the associations between the independent variables and youths’ excessive gambling in non-clinical samples. The participants were recruited from research panels administrated by Dynata. There were no missing data. The ethics committee of the Tampere region stated in December 2016 that the study included no ethical issues.

### 2.2. Measures

Excessive gambling was measured using the South Oaks Gambling Screen (SOGS), which is based on the diagnostic criteria of pathological gambling [38]. The participants were asked to assess whether they had experienced gambling-related problems, such as gambling more than intended, arguments with people about gambling, and borrowing money from multiple sources during the past 12 months. The scale contains 20 binary-scored items, giving a range of 0 to 20. The scale had good internal consistency. Cronbach’s alpha (α) was 0.89 in Finland, 0.90 in the United States, 0.80 in Spain, and 0.68 in South Korea. To minimise possible biases in estimates of excessive gambling that might have resulted from arbitrary categorization and the use of low cut-off points [39,40], the SOGS was used as a continuous variable. Higher values indicated higher levels of gambling problems.

Psychological distress was measured with the 12-item General Health Questionnaire (GHQ-12) which is a widely used screener of mental health problems [41]. The questionnaire had items assessing general factors of well-being, such as concentration, enjoyment, self-confidence, and happiness. Cronbach’s α was 0.88 in Finland, the United States, and South Korea, and it was 0.86 in Spain. The instrument was scored using bimodal scoring (0-0-1-1) for all 12 items, giving a range between 0 and 12. Higher values indicated higher psychological distress.

Loneliness was measured using the 3-item Loneliness Scale, which is a shorter but equally usable version of the original UCLA Loneliness Scale [42]. Its Cronbach’s α was 0.83 in Finland, 0.82 in the United States, 0.81 in Spain, and 0.84 in South Korea. The participants were asked how often they felt a lack of companionship, left out, and isolated from others. The response scale was 1 (hardly ever), 2 (some of the time), and 3 (often). The items were summed up and then divided by 3 to acquire average scores with values ranging from 1 to 3. Higher values indicated a higher sense of loneliness.

Sense of mastery was measured using the 7-item Pearlin Mastery Scale [30]. The participants were asked to assess how strongly they agreed or disagreed with statements about their control over life events and problems (e.g., I have little control over the things that happen to me) using a scale ranging from 1 (strongly agree) to 4 (strongly disagree). To unify the scoring with other variables, the first five items were inverted, after which a sum variable was created and divided by 7 to acquire average scores with values in the range of 1–4. Thus, higher values indicated a lower sense of mastery. Cronbach’s α values were 0.79 in Finland, 0.76 in the United States, 0.72 in Spain, and 0.71 in South Korea.

Age and gender were used as demographic control variables.

### 2.3. Analyses

**Figure 1:**
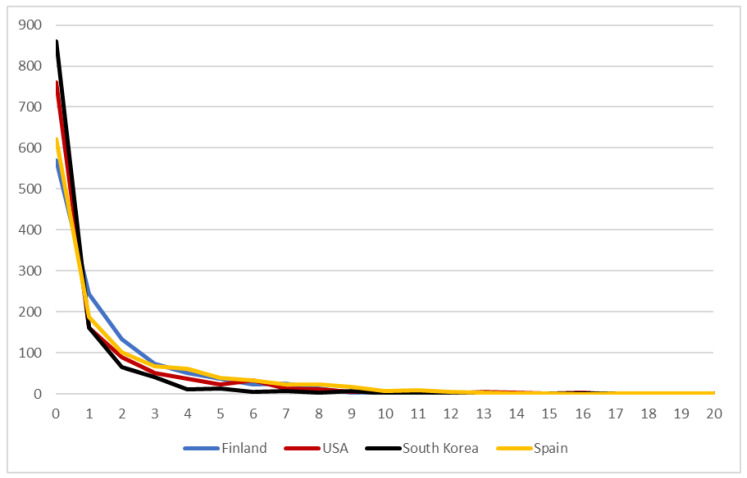
Distribution of excessive gambling in Finnish, U.S., South Korean, and Spanish data, as measured by the SOGS.

Analyses were conducted with Stata 16 statistical software by StataCorp, College Station, TX, USA. Several multivariate analysis methods were used to examine the associations between independent variables and the dependent variable. Although the sample size was considerably high, the distributions were moderately to highly skewed, which could result in biased estimates. This skewness applied especially to the SOGS (see Figure 1). Thus, the normality of the curves could not be assumed. Zero-inflated negative binomial regression (ZINB) analyses were conducted to account for overdispersion and excess zeroes. According to Yang et al. [43], ZINB models perform consistently well in such situations over alternatives. By analysing excess zeroes separately using odds ratios (ORs), ZINB gave more proper estimates of the effects of loneliness, mastery, and psychological distress on excessive gambling. These effects, in turn, were analysed using incidence rate ratios (IRRs), which are typically used to analyse count variables, such as those described here.

In addition to ZINB regression analyses, generalized structural equation models based path analyses were used to examine mediation between the dependent variable and independent variables. Psychological distress was positioned as a mediating variable, with loneliness and low sense of mastery as independent variables. The SOGS was the dependent variable in all analyses.

## 3. Results

Gambling was prevalent in all four country samples, but there were some minor differences in the distribution of SOGS scores (see Figure 1). In all countries, most respondents did not report experiencing problems related to excessive gambling. This was especially true in South Korea, where almost two-thirds of the respondents had a SOGS score of 0, whereas in Finland and Spain, only half of the respondents had similar scores. The highest rate of respondents who reported at least one gambling-related problem was observed in Finland (*n* = 631). However, the severity of excessive gambling was highest among Spanish respondents when a cut-off of 4 or more problems was crossed, and the differences evened out only in the proportion exceeding a score of 13 or more problems.

The results of ZINB analyses are reported in Table 1. Similarities and differences existed among the four countries with varying significance, especially in terms of excess zeroes, in other words, young people showing no excessive gambling. We found that distress was associated with a lack of excessive gambling only in the United States. In Finland, the United States, and South Korea, lack of excessive gambling was more common among younger respondents. Lack of excessive gambling was also more common among women in Finland and Spain.

Regarding the presence of excessive gambling in Table 1, only low sense of mastery and gender showed consistent significance in all countries. Rate ratios also varied between countries. The rate ratios for low sense of mastery varied from 1.31 (95% CI 1.02–1.67) in the U.S. data to 2.10 (95% CI 1.48–2.96) in the South Korean data, with the data from Finland (IRR: 1.56; 95% CI 1.24–1.96) and Spain (IRR: 1.39; 95% CI 1.10–1.74) falling in between. Thus, for every increase in low sense of mastery, the increase in SOGS scores can be expected to be 31 per cent in the United States, 39 per cent in Spain, 56 per cent in Finland, and 110 per cent in South Korea. Being male increased the SOGS scores by 69 per cent in Spain, 81 per cent in the United States, 101 per cent in Finland, and 185 per cent in South Korea.

In contrast, loneliness was a significant predictor of excessive gambling only in South Korea (IRR: 1.65; 95% CI 1.23–2.22) and Spain (IRR: 1.49; 95% CI 1.24–1.79), psychological distress was a significant predictor only in Finland (IRR: 1.06; 95% CI 1.02–1.10) and the United States (IRR: 1.07; 95% CI 1.02–1.12), and age was a significant predictor only in South Korea (IRR: 0.93; 95% CI 0.89–0.97). Here, every increase in loneliness showed a 65 per cent increase in excessive gambling in South Korea and a 49 per cent increase in Spain. Every increase in psychological distress showed an approximately 6–7 per cent increase in excessive gambling in Finland and the United States. Lastly, the relationship between age and excessive gambling was inverse in South Korea, meaning that every increase in age decreased excessive gambling by seven per cent.

**Figure 2:**
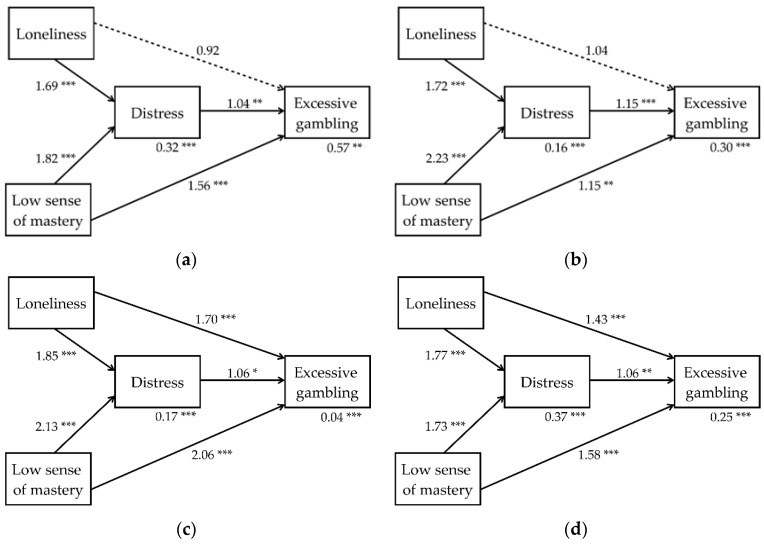
(**a**) Path model, Finnish data. The effect of loneliness and low sense of mastery on excessive gambling, with psychological distress as a mediating variable. (**b**) Path model, U.S. data. The effect of loneliness and low sense of mastery on excessive gambling, with psychological distress as a mediating variable. (**c**) Path model, South Korean data. The effect of loneliness and low sense of mastery on excessive gambling, with psychological distress as a mediating variable. (**d**) Path model, Spanish data. The effect of loneliness and low sense of mastery on excessive gambling, with psychological distress as a mediating variable. Values are expressed as rate ratios. * *p* < 0.05; ** *p* < 0.01; *** *p* < 0.001.

Further analyses (Figure 2a–d, Table 2a–d) showed the direct, indirect, and total effects of loneliness and low sense of mastery on excessive gambling, with psychological distress as the mediating variable. The effects were similar to previous analyses, as low sense of mastery was the strongest overall predictor of the severity of excessive gambling. Loneliness had a strong, significant direct and total effect in South Korea and Spain but not in Finland and the United States. However, the indirect effect was significant in all four countries. Psychological distress had a fairly low direct effect on excessive gambling and was only a partial mediator for the indirect effects of loneliness and low sense of mastery on excessive gambling.

## 4. Discussion

In this article, we aimed to examine how psychological distress mediates the associations between sense of mastery and loneliness and the severity of excessive gambling in cross-country data. Cross-country analyses showed that low sense of mastery was a consistently significant predictor of the severity of excessive gambling in all countries, both directly and indirectly, with psychological distress as a partial mediator. By contrast, loneliness predicted excessive gambling indirectly in all countries, but direct effects were found only in the South Korean and Spanish data. Moreover, indirect effects were not strong compared with direct effects, probably because psychological distress did not have a strong association with excessive gambling.

Low sense of mastery and loneliness had significant effects on psychological distress, which is in line with former research. Indeed, low sense of mastery is a likely stressor, so it contributes to the amount of distress a person might have [33]. Similarly, loneliness has been found to activate neuroendocrine stress mechanisms in both animal and human studies [27]. As discussed earlier, perceived loneliness varies in quality and quantity [20], and it is experienced uniquely in different social and normative environments. Thus, in collectivistic and socially cohesive societies such as Spain and South Korea, the role of loneliness—probably coming from a lack of satisfying relationships—might be stronger in addictive behaviours such as excessive gambling.

The low effect of psychological distress on the severity of excessive gambling was surprising, considering how closely psychiatric disorders are associated with excessive gambling [15,21]. One possible explanation for this result could be that there are multiple ways to attempt to cope with distress, and gambling is not among the most attractive alternatives if one does not already have a strong predisposition towards gambling.

In this cross-country study, the results provide insight into how different social and cultural environments might affect the psychosocial factors behind distress or excessive gambling. For instance, strict gambling regulations and overall negative gambling attitudes in South Korea might explain the overall lower excessive gambling levels among South Korean participants [44], whereas in Finland, different forms of gambling are widely available, making gambling a relatively easy and accessible habit [2]. However, male gender and sense of mastery predicted the severity of excessive gambling in all countries, suggesting that these factors may not be as country-dependent as other factors are. Because studies have linked trait impulsivity with excessive gambling [45,46], in future studies, it might be useful to consider whether sense of mastery is involved in these kinds of associations in some way. It is also worthwhile to recognize that, although gambling might be potentially harmful, psychosocial difficulties in life might also contribute to the development of excessive and harmful behaviours [9,10], even though the majority of people do not seem to form this kind of harmful relationship with gambling.

Our study had some limitations. First, no causal relationships can be established because of the cross-sectional nature of the data, and all suggested causalities are purely theoretical. Although our analyses provide some theoretical evidence for psychological distress as a mediating variable, this needs to be verified in longitudinal settings. Second, because of the complexity of human behaviour, the variables in our analyses may have more complicated and reciprocal relationships than we can provide here. Third, the data are not targeted exclusively at those who experience harm or even for those who gamble. The associations might be stronger in these groups. Finally, self-reported data are susceptible to pressure to provide socially desired answers, particularly in terms of stigmatized phenomena such as excessive gambling; however, it can be expected that the use of an anonymous online survey makes this bias less likely to occur compared with less anonymous situations.

This study showed some evidence for the effects of psychological distress on excessive gambling. Our study contributed to the theoretical discussion on the role of psychological distress in excessive gambling by scrutinizing how underlying factors such as loneliness and sense of mastery relate to distress and gambling. Loneliness and low sense of mastery can be stressful and harmful to well-being. Impairments in psychosocial well-being can influence various harmful behaviours, but they do not necessarily lead to excessive gambling. However, psychological distress can increase the severity of excessive gambling among those who gamble. Prevention and intervention strategies should focus on recognizing and improving young individuals’ overall well-being. Reinforcing youths’ sense of mastery could be particularly beneficial in the prevention and treatment of gambling problems. For instance, educational programs focused on teaching young individuals how to build and strengthen their sense of mastery through expectation management and resilience might be beneficial and help them maintain overall well-being.

## Figures and Tables

**Table 1:** Zero-inflated negative binomial regression models explaining the severity of excessive gambling and excess zeroes (no excessive gambling). * p < 0.05; ** p < 0.01; *** *p* < 0.001.

	Finland			United States			South Korea			Spain		
*Excessive gambling*	log(b)	IRR	Robust SE	log(b)	IRR	Robust SE	log(b)	IRR	Robust SE	log(b)	IRR	Robust SE
Distress	0.05 **	1.06 **	0.02	0.07 **	1.07 **	0.02	0.04	1.04	0.03	0.04	1.04	0.03
Loneliness	0.01	1.01	0.12	−0.10	0.90	0.12	0.50 **	1.65 **	0.15	0.40 ***	1.49 ***	0.09
Low sense of mastery	0.44 ***	1.56 ***	0.12	0.27 *	1.31 *	0.13	0.74 ***	2.10 ***	0.18	0.33 **	1.39 **	0.12
Age	−0.03	0.98	0.02	0.04	1.04	0.02	−0.07 **	0.93 **	0.02	0.01	1.01	0.02
Male gender	0.70 ***	2.01 ***	0.11	0.59 ***	1.81 ***	0.13	1.05 ***	2.85 ***	0.15	0.53 ***	1.69 ***	0.11
*No excessive gambling*	log(b)	OR	Robust SE	log(b)	OR	Robust SE	log(b)	OR	Robust SE	log(b)	OR	Robust SE
Distress	−0.03	0.97	0.10	−0.98 ***	0.37 ***	0.25	−2.12	0.12	1.66	−0.10	0.90	0.07
Loneliness	−0.03	0.98	0.57	−0.08	0.93	0.48	−1.26	0.03	1.15	0.28	1.32	0.24
Low sense of mastery	0.49	1.62	0.65	−0.16	0.85	0.38	0.97	2.63	0.77	−0.22	0.80	0.30
Age	−0.32 **	0.72 **	0.12	−0.33 *	0.72 *	0.14	0.13	1.14	0.12	−0.24 **	0.79 **	0.08
Male gender	−1.28 **	0.28 **	0.48	−0.04	0.96	0.64	0.46	1.59	1.03	−0.88 ***	0.41 ***	0.23
(/ln)alpha	0.03	1.03		0.79 ***	2.21 ***		1.00 ***	2.73 ***		−0.08	0.93	
Wald 0.066 χ^2^: (5)	68.14			38.23			118.28			58.90		
Max. likelihood R^2^	0.13			0.14			0.12			0.16		
Cragg & Uhler’s R^2^	0.13			0.15			0.14			0.16		
McFadden’s Adj. R^2^	0.03			0.04			0.05			0.04		

**Table 2:** Direct and indirect effects of loneliness and low sense of mastery, along with the direct effect of psychological distress on excessive gambling. (**a**) Finnish data. (**b**) U.S. data. (**c**) South Korean data. (**d**) Spanish data. Values are expressed as log(b). * *p* < 0.05; ** *p* < 0.01; *** *p* < 0.001; bootstrap: 5000.

*Excessive gambling*		Direct Effect	Robust SE	Indirect Effect	Bootstrap SE	Total Effect	Bootstrap SE
(a)	Distress	0.04 **	0.02	-	-	0.04 **	-
	Loneliness	−0.08	0.09	0.02 **	0.01	−0.06	0.09
	Low sense of mastery	0.44 ***	0.09	0.03 **	0.01	0.47 ***	0.09
(b)	Distress	0.14 *	0.02	-	-	0.14 *	-
	Loneliness	0.04	0.11	0.08 *	0.01	0.11	0.11
	Low sense of mastery	0.35 *	0.12	0.11 *	0.02	0.46 *	0.12
(c)	Distress	0.06 *	0.02	-	-	0.06 *	-
	Loneliness	0.53 ***	0.13	0.04 *	0.02	0.57 ***	0.13
	Low sense of mastery	0.73 ***	0.17	0.04 *	0.02	0.77 ***	0.17
(d)	Distress	0.06 **	0.02	-	-	0.06 **	-
	Loneliness	0.36 ***	0.09	0.03 **	0.01	0.39 ***	0.09
	Low sense of mastery	0.46 ***	0.11	0.03 **	0.01	0.49 ***	0.10

## Data Availability

YouGamble 2017–Finnish Data are publicly available in the Finnish Social Science Data Archive (http://urn.fi/urn:nbn:fi:fsd:T-FSD3399) (accessed on 26 June 2021). Data from the United States, South Korea, and Spain will be made publicly available in the Finnish Social Science Data Archive during 2021. The data are available from the corresponding author (A.O.) with a reasonable request.

## References

[1] Weinstock J. (2018). Call to action for gambling disorder in the United States. Addiction.

[2] Sulkunen P., Babor T.F., Cisneros Ornberg J., Egerer M., Hellman M., Livingstone C., Marionneau V., Nikkinen J., Orford J., Room R. (2019). Setting Limits: Gambling, Science, and Public Policy.

[3] Derevensky J.L., Gilbeau L., Heinz A., Romanczuk-Seiferth N., Potenza M.N. (2019). Preventing Adolescent Gambling Problems. Gambling Disorder.

[4] Delfabbro P., King D.L., Derevensky J.L. (2016). Adolescent Gambling and Problem Gambling: Prevalence, Current Issues, and Concerns. Curr. Addict. Rep..

[5] Calado F., Alexandre J., Griffiths M.D. (2017). Prevalence of Adolescent Problem Gambling: A Systematic Review of Recent Research. J. Gambl. Stud..

[6] Orford J. (2011). An Unsafe Bet? The Dangerous Rise of Gambling and the Debate We Should Be Having.

[7] Petry N.M., Blanco C., Auriacombe M., Borges G., Bucholz K., Crowley T.J., Grant B.F., Hasin D.S., O’Brien C. (2014). An Overview of and Rationale for Changes Proposed for Pathological Gambling in DSM-5. J. Gambl. Stud..

[8] Rantala V., Sulkunen P. (2012). Is pathological gambling just a big problem or also an addiction?. Addict. Res. Theory.

[9] Alexander B.K. (2008). The Globalization of Addiction.

[10] Blaszczynski A., Nower L. (2002). A pathways model of problem and pathological gambling. Addiction.

[11] Baumeister R.F., Leary M.R. (1995). The Need to Belong: Desire for Interpersonal Attachments as a Fundamental Human Motivation. Psychol. Bull..

[12] Ryan R.M., Deci E.L. (2017). Self-Determination Theory: Basic Psychological Needs in Motivation, Development, and Wellness.

[13] Lane W., Sacco P., Downton K., Ludeman E., Levy L., Tracy J.K. (2016). Child maltreatment and problem gambling: A systematic review. Child Abus Negl..

[14] Moustafa A.A., Parkes D., Fitzgerald L., Underhill D., Garami J., Levy-Gigi E., Stramecki F., Valikhani A., Frydecka D., Misiak B. (2018). The relationship between childhood trauma, early-life stress, and alcohol and drug use, abuse, and addiction: An integrative review. Curr. Psychol..

[15] Dowling N.A., Cowlishaw S., Jackson A.C., Merkouris S.S., Francis K.L., Christensen D.R. (2015). Prevalence of psychiatric co-morbidity in treatment-seeking problem gamblers: A systematic review and meta-analysis. Aust. N. Z. J. Psychiatry.

[16] Suomi A., Dowling N.A., Jackson A.C. (2014). Problem gambling subtypes based on psychological distress, alcohol abuse and impulsivity. Addict. Behav..

[17] Ciccarelli M., Griffiths M.D., Nigro G., Cosenza M. (2016). Decision making, cognitive distortions and emotional distress: A comparison between pathological gamblers and healthy controls. J. Behav. Ther. Exp. Psychiatry.

[18] Knaebe B., Knaebe B., Rodda S.N., Rodda S.N., Hodgins D.C., Hodgins D.C. (2019). Behaviour Change Strategies Endorsed by Gamblers Subtyped by Psychological Distress, Risky Alcohol Use, and Impulsivity. J. Gambl. Stud..

[19] Nigro G., D’Olimpio F., Ciccarelli M., Cosenza M. (2019). The fuzzy future: Time horizon, memory failures, and emotional distress in gambling disorder. Addict. Behav..

[20] Cacioppo J.T., Hawkley L.C., Crawford L.E., Ernst J.M., Burleson M.H., Kowalewski R.B., Malarkey W.B., Van Cauter E., Berntson G.G. (2002). Loneliness and health: Potential mechanisms. Psychosom Med..

[21] Castrén S., Basnet S., Salonen A.H., Pankakoski M., Ronkainen J.-E., Alho H., Lahti T. (2013). Factors associated with disordered gambling in Finland. Subst. Abus Treat. Prev. Policy.

[22] Sirola A., Kaakinen M., Savolainen I., Oksanen A. (2019). Loneliness and online gambling-community participation of young social media users. Comput. Human Behav..

[23] Mahapatra S. (2019). Smartphone addiction and associated consequences: Role of loneliness and self-regulation. Behav. Inf. Technol..

[24] Savolainen I., Oksanen A., Kaakinen M., Sirola A., Paek H.J. (2020). The role of perceived loneliness in youth addictive behaviors: Cross-national survey study. J. Med. Internet Res..

[25] Yao M.Z., Zhong Z. (2014). Loneliness, social contacts and Internet addiction: A cross-lagged panel study. Comput. Human Behav..

[26] Mund M., Freuding M.M., Möbius K., Horn N., Neyer F.J. (2020). The Stability and Change of Loneliness Across the Life Span: A Meta-Analysis of Longitudinal Studies. Personal Soc. Psychol. Rev..

[27] Cacioppo J.T., Cacioppo S., Capitanio J.P., Cole S.W. (2015). The neuroendocrinology of social isolation. Annu. Rev. Psychol..

[28] Heu L., van Zomeren M., Hansen N. (2020). Does loneliness thrive in relational freedom or restriction? The culture-loneliness framework. Rev. Gen. Psychol..

[29] Orford J. (2013). Power, Powerlessness and Addiction.

[30] Pearlin L.I., Schooler C. (1978). The Structure of Coping. J. Health Soc. Behav..

[31] Conger K.J., Williams S.T., Little W.M., Masyn K.E., Shebloski B. (2009). Development of mastery during adolescence: The role of family problem-solving. J. Health Soc. Behav..

[32] Bandura A., Pastorelli C., Barbaranelli C., Caprara G.V. (1999). Self-Efficacy Pathways to Childhood Depression. J. Pers. Soc. Psychol..

[33] Dalgard O.S., Mykletun A., Rognerud M., Johansen R., Zahl P.H. (2007). Education, sense of mastery and mental health: Results from a nation wide health monitoring study in Norway. BMC Psychiatry.

[34] Pryce C.R., Azzinnari D., Spinelli S., Seifritz E., Tegethoff M. (2011). Meinlschmidt, G. Helplessness: A systematic translational review of theory and evidence for its relevance to understanding and treating depression. Pharmacol. Ther..

[35] Bozzato P., Longobardi C., Fabris M.A. (2020). Problematic gambling behaviour in adolescents: Prevalence and its relation to social, self-regulatory, and academic self-efficacy. Int. J. Adolesc. Youth.

[36] Williams R.J., Volberg R.J., Stevens R.M.G. The Population Prevalence of Problem Gambling: Methodological Influences, Standardized Rates, Jurisdictional Differences, and Worldwide Trends. http://hdl.handle.net/10133/3068.

[37] Oksanen A., Sirola A., Savolainen I., Koivula A., Kaakinen M., Vuorinen I., Zych I., Paek H.-J. (2021). Social ecological model of problem gambling: A cross-national survey study of young people in the united states, south korea, spain, and finland. Int. J. Environ. Res. Public Health.

[38] Lesieur H.R., Blume S.B. (1987). The South Oaks Gambling Screen (SOGS): A new instrument for the identification of pathological gamblers. Am. J. Psychiatry.

[39] Stinchfield R. (2002). Reliability, validity, and classification accuracy of the South Oaks Gambling Screen (SOGS). Addict. Behav..

[40] Goodie A.S., MacKillop J., Miller J.D., Fortune E.E., Maples J., Lance C.E., Campbell W.K. (2013). Evaluating the South Oaks Gambling Screen With DSM-IV and DSM-5 Criteria: Results From a Diverse Community Sample of Gamblers. Assessment.

[41] Goldberg D.P., Gater R., Sartorius N., Ustun T.B., Piccinelli M., Gureje O., Rutter C. (1997). The validity of two versions of the GHQ in the WHO study of mental illness in general health care. Psychol. Med..

[42] Hughes M.E., Waite L.J., Hawkley L.C., Cacioppo J.T. (2004). A short scale for measuring loneliness in large surveys: Results from two population-based studies. Res Aging.

[43] Yang S., Puggioni G., Harlow L.L., Redding C.A. (2017). A comparison of different methods of zero—Inflated data analysis and an application in health surveys. J. Mod. Appl. Stat. Methods.

[44] Williams R.J., Williams R.J., Lee C.-K., Lee C.-K., Back K.J., Back K.J. (2013). The prevalence and nature of gambling and problem gambling in South Korea. Soc. Psychiatry Psychiatr. Epidemiol..

[45] Hodgins D.C., Holub A. (2015). Components of Impulsivity in Gambling Disorder. Int. J. Ment. Health Addict..

[46] Ioannidis K., Hook R., Wickham K., Grant J.E., Chamberlain S.R. (2019). Impulsivity in Gambling Disorder and problem gambling: A meta-analysis. Neuropsychopharmacology.

